# Butyrate induces profound changes in gene expression related to multiple signal pathways in bovine kidney epithelial cells

**DOI:** 10.1186/1471-2164-7-234

**Published:** 2006-09-14

**Authors:** Robert W Li, CongJun Li

**Affiliations:** 1 Bovine Functional Genomics Laboratory, Animal and Natural Resources Institute, United States Department of Agriculture-Agricultural Research Service, Beltsville, MD 20705, USA; 2 Growth Biology Laboratory, Animal and Natural Resources Institute, United States Department of Agriculture-Agricultural Research Service, Beltsville, MD 20705, USA

## Abstract

**Background:**

Global gene expression profiles of bovine kidney epithelial cells regulated by sodium butyrate were investigated with high-density oligonucleotide microarrays. The bovine microarray with 86,191 distinct 60mer oligonucleotides, each with 4 replicates, was designed and produced with Maskless Array Synthesizer technology. These oligonucleotides represent approximately 45,383 unique cattle sequences.

**Results:**

450 genes significantly regulated by butyrate with a median False Discovery Rate (FDR) = 0 % were identified. The majority of these genes were repressed by butyrate and associated with cell cycle control. The expression levels of 30 selected genes identified by the microarray were confirmed using real-time PCR. The results from real-time PCR positively correlated (R = 0.867) with the results from the microarray.

**Conclusion:**

This study presented the genes related to multiple signal pathways such as cell cycle control and apoptosis. The profound changes in gene expression elucidate the molecular basis for the pleiotropic effects of butyrate on biological processes. These findings enable better recognition of the full range of beneficial roles butyrate may play during cattle energy metabolism, cell growth and proliferation, and possibly in fighting gastrointestinal pathogens.

## Background

The most common short-chain fatty acids (SCFA) are natural microbial fermentation products in the gastrointestinal tract. SCFA, including propionic, butyric and valeric acids, each with three, four and five carbons, respectively, contribute to the energy balance of all mammalian species [1,2]. The major sources of these carbohydrates are hemicelluloses and fiber, which consists of plant cell wall polysaccharides such as cellulose and pectins [1]. In ruminants, SCFA are a major energy source and contribute up to 70% of their energy requirements [2]. Beyond their nutritional impact, SCFA, especially butyrate, have a multitude of cellular regulatory effects that modulate cell differentiation, proliferation, and motility. All three major components of SCFA induce apoptosis and inhibit cell proliferation, however, butyrate has the most potent effect [3,4]. Roles for butyrate have been established in cell differentiation, proliferation, motility and in particular induction of cell cycle arrest and apoptosis [5-7]. Apoptosis is a genetically regulated cellular suicide mechanism that plays a crucial role in development and in the defense of homeostasis of animals [8]. The mechanism(s) by which butyrate induces cellular differentiation and suppresses growth has not been elucidated. Similarly, the mechanism through which butyrate and other short-chain fatty acids induce the cell cycle regulatory and apoptotic effects and the mechanism by which the decision between cell death and survival is arbitrated are poorly understood [9-12].

In a previous study [13], potential biological roles of butyrate were investigated using the established Madin-Darby bovine kidney epithelial cell line (MDBK). The study focused on determining whether normal bovine cells in a standard cell culture condition were sensitive to the growth inhibitory effects of butyrate. The data suggested that sodium butyrate (SB) could induce apoptosis and cell cycle arrest in MDBK cells. Up to 38% of cells became apoptotic after 24 hours of treatment with 10 m*M *of butyrate. Butyrate also blocked the surviving cells at two distinct stages, G1 and M/G2. However, more studies are needed to better understand the relationship between butyrate and alterations in the expression of genes involved in cell cycle, apoptosis, and transcriptional regulation. Recent advances in high-throughput genomic tools such as microarray technology allowed us to examine the genome-wide effects of sodium butyrate on MDBK cells.

## Results

### Butyrate Induces Cell Cycle Arrest and Hyperacetylation of Histone 3 in MDBK Cells

**Figure 1:**
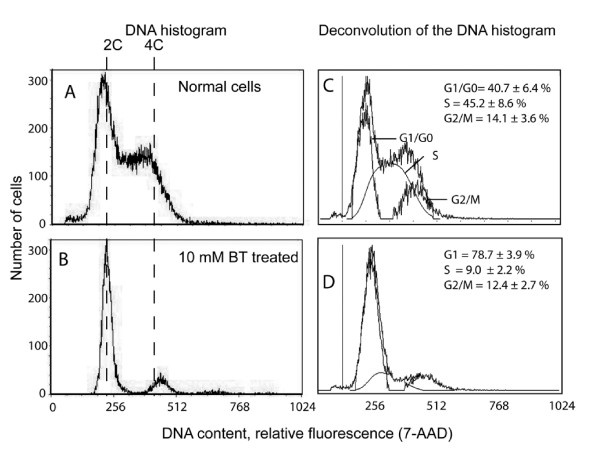
Cell cycle progressions determined by flow cytometry. Histogram plot of flow cytometry analysis of A: control cells; B: the cells treated by 10 m*M *sodium butyrate. Deconvolution of the DNA histogram using automated cell cycle analysis with 'Cylchred' software of C: control cells; and D: the cells treated by 10 m*M *sodium butyrate. Insert: Mean ± SEM (n = 3 per treatment; **: *P *< 0.01. G1/G0: cells in G1/G0 cell cycle phases; S: cells in S cell cycle phase; M/G2: cells in M/G2 cell Cycle phases). 2C and 4C: two and four copies of DNA content respectively.

**Figure 2:**
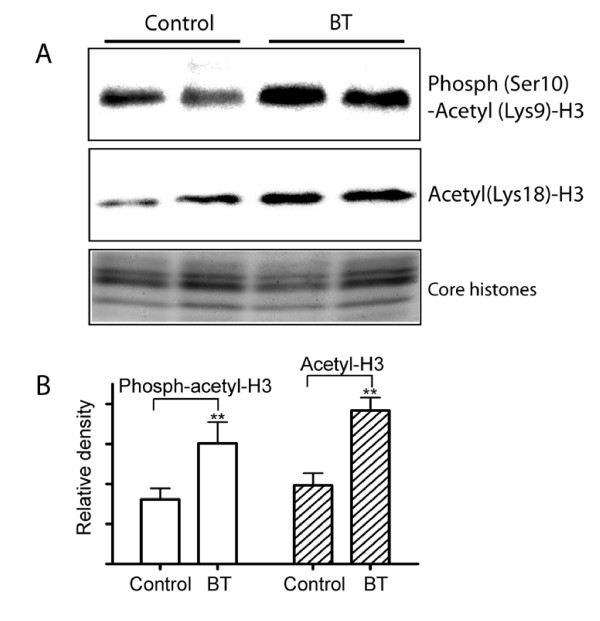
Butyrate induced hyperacetylation of histone 3 (H3). This figure is representative of three experiments (n = 3). A: Protein from different samples was separated by SDS PAGE on two identical 4 to 20% polyacrylamide gradient gels. One gel was stained with SimpleBlue (Invitrogen) and one was transferred to a membrane for Western blotting with monoclonal anti- acetyl-phospho H3 and anti- acetyl H3 antibodies; B: Western Blots from three experiments were quantified with NIH Image software. The relative densities were measured and corrected with the protein density. H3: histone 3. **: *P *< 0.01.

We previously reported that butyrate induced cell cycle arrest in MDBK cells [13]. Prior to microarray analysis, the butyrate induced cell cycle arrest was reconfirmed. As shown in Figure 1, after butyrate treatment for 24 h, cell population profiles changed significantly. In the surviving cell population there was a significant increase in the number of cells in G1 (cells with 2C DNA contents) whereas those in S phase (cells falling between the 2C and 4C DNA contents) were decreased. This result confirmed our prior observation that cells were arrested at the G1/S boundary and DNA replication was blocked by the butyrate treatment. We also confirmed accumulation of acetylated histone 3 (H3) due to the butyrate treatment (Fig. 2). H3 acetylation was selected as the marker for the accumulation of acetylated histones because H3 is one of the core histones (H2A, H2B, H3, and H4), and is highly conserved across a wide range of organisms. The antibody against the acetyl H3(lys18) is also readily available commercially. To determine whether these biochemical attributes of butyrate were also observed in MDBK cells, two specific antibodies, the monoclonal antibody against the acetyl H3 and the monoclonal antibody against acetyl-phospho H3, were used to evaluate the histone deacetylase inhibitory activity of butyrate. Histone deacetylase (HDAC) inhibitors induce the hyperacetylation of nucleosomal histones. Evidence suggested that H3 phosphorylation is restricted to a small fraction of highly acetylated H3 histones. H3 phosphorylation is cell cycle dependent and may be associated with induced FOS and MYC oncogenes. No evidence indicates H3 phosphorylation directly results from HADC inhibitory activities. As shown in Fig. 2, butyrate treatment induced not only accumulation of hyperacetylation of H3 (Lys18) but also phosphor (Ser10)-acetyl (Lys9) H3. The quantified relative densities from the Western Blot show approximately a 2-fold increase of acetylated H3 protein and 2-fold increase of phosphor-acetyl H3 (Fig. 2B). Therefore it is reasonable to assume the increase detected by the antibody against phosph(Ser10)-Acetyl (lys9)-H3 is mostly due to the acetylation of H3 induced by butyrate.

### Butyrate Induces Profound Changes in Gene Expression in MDBK Cells

In our previous study [13] and prior to the current microarray experiment, we monitored butyrate induced cell death and cell cycle arrest in MDBK cells in a time/dose-dependent manner using flow cytometry and Western blotting. For the purpose of this investigation and for determining the differential gene expression induced by butyrate, we selected a single dose proven to be able to generate maximum biological impacts in cell cycle arrest. Due to research budget constraints, we selected only a single time point as the initial global expression screening. We intend to verify the genes identified with the microarray in a time/dose-dependent manner using real-time RT-PCR in subsequent studies. We identified 450 genes significantly regulated by sodium butyrate at a very stringent false discovery rate (FDR) = 0% (see Additional file 1). However, when relaxing the stringency threshold to FDR = 10%, which should still be acceptable in most cases, there were 3662 genes significantly regulated (3662/45383 = 8%, data not shown). This percentage was consistent with a previous report [6] in which the authors used cDNA microarrays consisting of ~8000 sequences to demonstrate that approximately 7% of sequences assayed exhibited alteration by butyrate in human colon carcinoma cells.

### Cell Cycle Control

The single largest category of genes regulated by butyrate (103) was cell cycle control related (Table 1). Butyrate repressed the vast majority of these genes including cyclins, cyclin-dependent kinases, histone deacetylases, helicases, chromosomal structure proteins as well as kinesins. Ubiquitin conjugating enzyme E2 C was also down-regulated. However, Max interacting protein 1 (MXI1) was up-regulated 12.6-fold by butyrate.

### Apoptosis and Extracellular Matrix

Fourteen genes related to apoptosis and extracellular matrix (ECM) were significantly regulated by butyrate (Table 2). Generally, butyrate induced pro-apoptotic genes and repressed anti-apoptotic genes. Inhibin, beta A (INHBA) and adrenomedullin were among the induced genes while apoptosis inhibitors such as survivin and FAIM were down-regulated. Interestingly, Insulin-like growth factor 2 (IGF2) was up-regulated while IGF binding proteins 4 and 6 were down-regulated. Collagens, such as COL5A2 and COL3A1, and extracellular matrix protein Spondin 1 were repressed by butyrate while tissue inhibitor of metalloproteinase 2 (TIMP2) was significantly up-regulated.

### Real-Time RT PCR

Thirty genes that represent different expression levels and functional classes were selected for real-time RT-PCR confirmation (Table 3). The real-time PCR data generally confirmed the microarray analysis. Linear regression analysis demonstrated a strong positive correlation between the two technological platforms with R = 0.867.

## Discussion

Cell cycle regulatory and apoptotic effects of butyrate and other short-chain fatty acids at the cellular and molecular levels in normal bovine cells have not been studied thus far but would serve as a principle launch point to validate the need for further study of these phenomena in cattle. Furthermore, utilization of particular signaling pathways in regulating cellular function or inducing gene expression appears to be dependent on, among other factors, the type of stimulus and cell examined. In our previous study, an important question asked and answered was whether normal bovine cells in a standard cell culture condition are sensitive to the growth inhibitory effects of butyrate[13]. In this follow-up study, we utilize microarray technique to examine the genome-wide effects of sodium butyrate on MDBK cells as an important component of our efforts to understand the mechanisms of this phenomenon. Our data presented in this report indicate that the effects of butyrate are mediated through coordinated changes in gene expression that are the outcome of interactions between transduction pathways. We identified 450 genes significantly regulated by sodium butyrate at a very stringent false discovery rate (FDR) = 0%. However, since many of the genes have no direct links to the cell cycle arrest or apoptosis, their involvement in these biological effects certainly warrants more investigation. Therefore, instead of speculating, our discussion is concentrated primarily in cell cycle and apoptosis.

Sodium butyrate (SB) exerts a very broad range of effects on many biological pathways via its inhibitory ability on HDAC. SB is a potent inducer of a G1 cell cycle arrest. It also provokes apoptosis by activating both the death receptor and intrinsic apoptotic pathway. It regulates the cell cycle via down-regulation of cyclins and activation of CDK [14]. In addition, it was reported that butyrate modulates host immune responses by activating neutrophils [15] and enhancing IL-4-dependent IgE production [16]. Butyrate has immune suppression [17] and anti-inflammatory properties, in part by suppressing nuclear factor NF-κB activity [18-20]. Regulation of enzymes involved in cytoskeleton and cell membranes by butyrate has also been reported [21]. The majority of its effects directly result from HDAC inhibition. However, butyrate appears to be involved in signal transduction via its own receptor GPR41, resulting in inosityl 1,4,5-triphosphate generation, intracellular Ca^2+ ^release, ERK1/2 activation, and inhibition of cAMP accumulation [22]. Because of their ability to inhibit cell proliferation, HDAC inhibitors (HDI) including butyrate have been extensively exploited as anti-tumor agents [23]. Butyrate appears to have pleiotropic effects on various biological processes. The genes we identified (see Additional file 1), which fall within a broad range of functional categories, appeared to provide the molecular basis for its pleiotropic effects.

Butyrate seems able to inhibit all class I HDACs [24]. It would be safe to assume that, like trichostatin A (TSA), butyrate can indeed inhibit the activities of HDAC8 and HDAC10, even though butyrate may have a different mechanism of action. Our studies suggested that butyrate indeed repressed histone deacetylase 8 (HDAC8) mRNA expression. The missing link is why this inhibition of enzymatic activities in turn down-regulates their own expression in mRNA levels. In mouse neural cells, it was observed that HDAC inhibitors affect the expression of HDACs themselves [18]. In these cells, both TSA and SB indeed elevated the expression of HDAC1, HDAC3, HDAC5 and HDAC6 whereas mRNA levels of HDAC 2 and HDAC7 did not change. The mRNA levels of HDAC8 and HDAC10 were not detectable in these cells. While the mechanism and biological relevance of HDI regulation of HDAC expression remains unclear, it appears that there indeed exists an auto-regulatory feedback loop to the expression of several HDACs after their activities are inhibited.

It appears that the effects of HDI such as TSA and SB on MMP (matrix metalloproteinase) expression are specific to cell types. In mouse 3T3 fibroblasts, TSA represses MMP2 expression [25], while in human colonic cells DHD/K12, MMP production is inhibited by butyrate [26]. In HT1080 tumor cells, both protein and mRNA levels of TIMP1, TIMP2, MMP2 and MMP9 are increased by butyrate treatment [27]. Based on their data of limited modulation of MMP by butyrate in human SW1116 colon cancer cells [28], Emenaker et al suggested that SCFA, such as those derived from dietary fiber, may protect against invasive colon cancer through stimulation of TIMP and inhibition of uPA (plasminogen activator, urokinase) activities rather than their effects on MMP activities. In the present study, we found that both TIMP2 and MMPs, such as MMP1, MMP9 and MMP13, were induced by butyrate. The expression levels of TIMP2 induced by butyrate are similar (10.82, 11.28, and 14.05 folds, respectively) as detected by the three sequences that represent this gene on the microarray [TIGR: TC260960, TIGR: TC289409 and TIGR: TC289410]. However, changes in MMP levels were not considered significant due to the high stringency cutoff used in this study (FDR = 0%). The net effect of this induction on both MMPs and their inhibitor (TIMP2) is still unclear. TIMP2 can promote apoptosis in an *in vivo *colorectal cancer model yet can protect B16 melanoma cells from apoptosis [29]. The elucidation of the mechanisms involved in controlling these distinctly opposing phenotypic effects of TIMP2 is of paramount importance.

Insulin-like growth factor binding proteins (IGFBP) modulate IGF action and regulate cell growth and apoptosis by preventing IGF from interacting with their own receptors. In our study, insulin-like growth factor (IGF-2) was upregulated by SB, which is consistent with other published data [30]. Our microarray and real-time RT-PCR results confirmed that IGFBP6, which has a 100-fold higher affinity for IGF2 than IGF1 [31], was down-regulated by butyrate. It seems paradoxical that while IGF2 is up-regulated significantly by SB, its highest affinity binding protein is down-regulated. Our results may suggest different functions of various IGFBP members in regulating apoptosis and cell cycle progression. It would be intriguing to see how IGFBP exert their actions in cell growth and apoptosis via an IGF-independent fashion.

Despite the fact that the effect of HDAC inhibitors such as SB on the expression of cell cycle regulatory genes, such as cyclins and cyclin-related kinase (CDK), were documented and a few attempts were made to use high-throughput approaches, such as microarrays [6], differential display [32] and SAGE, to study the effects of HDAC inhibitors on cell cycle control, the extent of the effect of these inhibitors on cell proliferation and cell cycle has not been fully realized in part due to limited gene representation on these microarrays [19,30,33,34]. Due to significant differences in gene representation, species, microarray platforms, and data analysis tools used in these studies, a direct comparison between our results and those published is seemingly difficult. In general, our results are in good agreement with published reports. For example, we detected ~8% of all genes were significantly regulated by butyrate in bovine MDBK cells, which is consistent with a previous report [6] in which the authors used cDNA microarray consisting of ~8000 sequences to demonstrate that approximately 7% of sequences assayed exhibited alteration by butyrate in human colon carcinoma cells. Down-regulation of cyclins, PCNA, CDKs, and upregulation of IGF2, MMPs, and TIMP2 were also confirmed by previous reports [19,34]. However, many genes, such as Aurora kinases, BUB1 and BUB1B, centromere proteins, kinesins, Max interacting protein 1 (MXI1), minichromosomal maintenance deficient proteins (MCMs), and spindle pole body components were not previously recognized to be regulated by SB. Our efforts in this study are among the first to systematically categorize the butyrate-regulated genes related with cell cycle control with a genome-wide approach in farm animals. While the vast majority of these genes are down-regulated, MXI1 is up-regulated. As a key component of the mitotic checkpoint, MXI1 binds with MAX to form a sequence-specific DNA-binding protein complex and acts as a transcriptional repressor. Up-regulation of MXI1 by SB could result in down-regulation of cyclins, which in turn negatively regulates centromere proteins.

Accumulation of cells with 2C and 4C DNA contents suggests inhibition by butyrate of cell cycle at G1 and M/G2 phases and suggests that a common responding element in genes responsive to the treatment of butyrate is required for progression of both phases G1 and M/G2. This observation is also consistent with the previous report that the inhibition of G1 progression by butyrate is not restricted to a specific mitogenic signaling pathway [35], but may also include the inhibitory effect on initiation of DNA replication. In this report, multiple genes such as minichromosome maintenance (MCM) proteins 2, 3, 4, 5, and 6, as well as Orc1 (Origin Recognition Complex largest subunit) are significantly down-regulated. The products of these genes has been shown to be rate limiting for initiation of DNA replication in eukaryotic cell lines and are essential for the assembly of the pre-replication complex (pre-RC) [36-38]. This finding indicates that in someway, butyrate treatment directly targets these genes and down regulates the genes that are essential for initiation of DNA replication. CDC2/Cdk1 and related cyclins are also significantly down-regulated. At least four roles have now been recognized for cyclin A activated Cdk1 protein kinases in regulating cell cycle events. First, Cdk2/cyclin A is responsible for activating pre-replication complexes at the beginning of S-phase in order to begin DNA synthesis [39]. Second, Cdk1/cyclin A inhibits assembly of new pre-replication complexes during S-phase [39] by inactivating Cdc6 [40]. Third, Cdk2/cyclin A is required for the G2 to M-phase transition [41]. Finally, Li and DePamphilis [14] recently revealed that Cdk1/cyclin A is required for preventing Orc1 in mammals or ORC in *Xenopus *from binding to chromatin during mitosis. Our results show that targeted destruction of cdc6 and cdc2/cdk1 may be involved in the apoptosis and cell cycle arrest induced by butyrate. They are consistent with the growing body of evidence suggesting that disruption of the coordination between regulation of DNA synthesis and cyclin-dependent kinase activity is an important feature of apoptosis. It is of importance and interest for us to understand the mechanism(s) of how butyrate targets these genes and causes these changes in gene expressions. However, it will require a great deal of efforts and certainly is out of the scope of this report.

Understanding the mechanism of butyrate in affecting apoptosis, cell proliferation and differentiation and especially the difference of its mode of action compared to other HDAC inhibitors will facilitate designing novel and more potent HDAC inhibitors that target specific cellular processes that are dysregulated in neoplastic cells. Dissection of the pathways regulated by butyrate will provide a basis for better utilization of its anti-tumor, anti-metastatic, immune-mediating, and anti-inflammatory properties. In addition, butyrate has been shown to up-regulate transcription levels of muslins, which are the major components of gastrointestinal mucosa considered to be the first line of defense against pathogens [42]. Because of its ability to affect functions of monocyte-derived dendritic cells and macrophage [17] and to activate neutrophils, butyrate and its related biological pathways could be manipulated to fight against gastrointestinal pathogens. Up-regulation of glutathione S-transferases [43,44], genes known to be involved in defense against oxidative stress, by butyrate provides evidence of a favorable modulation of toxicological defense systems. The results presented in this paper not only help to better dissect HDAC inhibition and mechanism in apoptosis and cell cycle control but also help to understand ruminant metabolism and physiology, which in turn could lead to improvement in energy efficiency of cattle.

## Conclusion

The present research identified 450 genes significantly regulated by sodium butyrate in bovine kidney epithelial cells. The genes related to multiple signal pathways such as cell cycle control and apoptosis were presented. The profound changes in gene expression elucidate the molecular basis for the pleiotropic effects of butyrate on biological processes. The results presented in this paper can provide clues on the mechanism of histone deacetylase inhibition by butyrate and resulting alterations in the expression of genes involved in cell cycle, apoptosis, and transcriptional regulation. Since butyrate functions as both a nutrient and signaling molecule regulating the cell growth and proliferation, these findings enable better recognition of the full range of roles butyrate may play during cattle energy metabolism, cell growth and proliferation.

## Methods

### Cell Culture and Treatments

The Madin-Darby bovine kidney epithelial cells (MDBK, American Type Culture Collection, Manassas, VA., Catalog No. CCL-22) were cultured in Eagle's minimal essential medium supplemented with 5% fetal bovine serum (Invitrogen, Carlsbad, CA) in 25 cm^2 ^flasks with medium renewal twice per week. Cell cultures were maintained in a water-jacked incubator with 5% CO_2 _at 37°C. Sub-cultivations were performed when cells attained 80 to 90 % confluence, according to the product information supplied by American Type Culture Collection. At approximately 50% confluence (during the exponential phase), the cells were treated with 10 mM of sodium butyrate for 24 h (Calbiochem, San Diego, CA. Three replicate flasks of cells for both treatment and control groups (a total of 6 samples) were used for the flow cytometry and microarray experiments. The harvested cells were snap frozen in liquid N2 and stored at -80°C until RNA extraction.

### Flow Cytometric Analysis of Cells

The detailed procedures were described in a previous publication [13]. Briefly, cells collected by trypsinization were washed and resuspended in PBS buffer. Two volumes of ice-cold 100% ethanol were added drop wise into tubes and mixed with cells in suspension by slow vertexing. After incubation with RNase I, cells were then stained with propidium iodide (PI). Measuring the fluorescence by flow cytometry provided a measure of the amount of PI taken up by the cells and, indirectly, the amount of DNA content. Cell DNA content was analyzed using a flow cytometer (FC500, Beckman Coulter, Palatine, IL) and collected data were analyzed using Cytomics RXP (Beckman Coulter). At least 10,000 cells per sample were analyzed.

### Preparation of Cell Extracts and Western Blot Analysis

Preparations of cells and cell extracts, SDS-PAGE and Western Blot analysis were described previously [13]. Briefly, the protein from different samples was separated by SDS PAGE on two identical 4 to 20% polyacrylamide gradient gels. One gel was stained with SimpleBlue (Invitrogen) and one was transferred to a membrane and probed with monoclonal anti- acetyl-phospho H3 and anti- acetyl H3 antibodies. The target bands on the Western Blots from three experiments were quantified with a NIH Image software. The relative densities were measured and corrected with the stained protein density.

### Isolation of Total RNA

Total RNA was extracted using Trizol following the manufacturer's recommendations (Invitrogen). Trace genomic DNA in the crude total RNA samples was removed by incubation with 4–10 units DNase I per 100 μg total RNA (Ambion, Austin, TX) at 37°C for 30 min. Total RNA was further purified using an RNeasy Mini kit (Qiagen, Valenica, CA). The concentration of the total RNA was determined using a NanoDrop ND-1000 spectrophotometer (NanoDrop Technologies, Rockland, DE) and RNA integrity was verified using a Bioanalyzer 1000 (Agilent, Palo Alto, CA).

### Generation of Biotin-labeled cRNA

Biotin-labeled cRNA was generated with a modified procedure of the Superscript Choice System (Invitrogen) for double-strand (ds) cDNA synthesis followed by *in vitro *transcription. Briefly, the 1^st ^strand cDNA was synthesized from 4.0 μg total RNA by 1.0 unit SuperScript II reverse transcriptase (Invitrogen) in the presence of 100 pmoles T7 promoter Oligo dT primer. After 2^nd ^strand synthesis, the DNA was purified with a DNA Clean & Concentrator-5 kit (Zymo Research, Orange, CA) and eluted with 8 to 16 μl of deionized (dd) H_2_O. The recovered ds cDNA was further concentrated down to 3 μl by a speed vacuum device. cRNA was synthesized with a MEGAscript *in vitro *Transcription kit (Ambion). The *in vitro *transcription reaction was carried out in a total volume of 23.0 μl consisting of 3.0 μl of ds cDNA, 2.3 μl 10X Ambion reaction buffer, 2.3 μl 10X Ambion T7 enzyme mix, and 15.4 μl NTP labeling mix (7.5 mM ATP, 7.5 mM GTP, 5.625 mM UTP, 5.625 mM CTP and 1.875 mM biotin-16-UTP and 1.875 mM biotin-11 CTP). The *in vitro *transcription reaction was incubated at 37°C for ~16 hours in a thermocycler. The cRNA was purified with an RNeasy mini kit (Qiagen). Generally, 40 to 60 μg of cRNA can be obtained from 4.0 μg of input total RNA. The size range of the cRNA, expected to be between 300 to 3000 bp with the maximum intensity centered at least 1000 bp, was verified using a Bioanalyzer 1000. The biotinylated cRNA was fragmented to 50 to 200 bp by heating cRNA in a buffer consisting of 40 mM Tris-acetate, pH 8.0, 100 mM potassium acetate, and 30 mM magnesium acetate at 95°C for 35 min.

### Oligonucleotide Microarray, Hybridization, Image Acquisition and Data Analysis

The bovine microarray platform used was described previously [45]. Briefly, a total of 86,191 unique 60mer oligonucleotides were designed and synthesized it *in situ *using photo deprotection chemistry [46]. Each unique oligonucleotide was repeated 4 times on the array (a total of ~340,000 features). These oligonucleotides represented 45,383 unique bovine sequences/genes, including 40,808 Tentative Consensus sequences (TCs) from TIGR *Bos taurus *gene index [47] and 4,575 singletons.

The microarrays were pre-hybridized with 1X MES hybridization buffer (100 mM MES, 1.0 M Na^+^, 20 mM EDTA, 0.01% Tween20), 40 μg herring sperm DNA and 200 μg acetylated BSA at 45°C for 15 min followed by hybridization with 10 μg denatured and fragmented cRNA per microarray at 45°C for 16 – 20 h with constant rotation. After hybridization, the microarrays were immediately washed extensively under non-stringent conditions (6× SSPE, 0.01% Tween20) at room temperature (RT) followed by a stringent wash (100 mM MES salt and free acid solution, 0.1 M Na^+^, 0.01% Tween20) at 45°C. After the final rinse with the non-stringent wash buffer, the microarrays were stained with 1× Stain buffer (100 mM MES, 1 M Na^+^, 0.05% Tween20, 50 mg/ml of BSA, and 1 mg/ml of Cy3-streptavidin) at RT for 25 min. The stain buffer was removed and the microarrays were rinsed once more with non-stringent wash buffer. The microarrays were immediately dried under a stream of argon gas and scanned using an Axon GenePix 4000B scanner (Molecular Devices Corp., Union City, CA) at 5 μM resolution. The data were extracted from the raw images using NimbleScan software (NimbleGen, Madison, WI). The control and butyrate treatment each had 3 replicates and a total of 6 microarrays were used in the experiment (GEO Accession GSE3970). The microarray data are available as accession GSE3970 in the Gene Expression Omnibus repository at the National Center for Biotechnology Information [48].

Relative signal intensities (log_2_) for each feature were generated using the Robust Multi-Array Average (RMA) algorithm [49,50]. The data were processed based on quantile normalization method [51] using the R package [52]. This normalization method aims to make the distribution of intensities for each array in a set of arrays the same. The method assumes that a quantile-quantile plot of two data vectors with the same distribution will have a straight diagonal line. The method performed better in dealing with bias and reducing variability across arrays compared to other methods [51]. The background-adjusted, normalized, and log transformed intensity values were then analyzed using the Significance Analysis of Microarrays method [53] with two-class unpaired design (SAM version 2.20 [54]). SAM is the most popular method for microarray analysis with 635 citations of the original publication as of October 2004 [55]. SAM ranks genes based on a modified *t*-test statistic. The unique features of SAM include implementing permutation testing, and the ability to estimate a global false discovery rate (FDR, an expected percentage of false positives among the claimed positives) and a gene error chance (*q*-value). A sequence was declared to be significant when it met a stringent median false discovery rate (FDR) cutoff at 0 % (see Additional file 1). A BLAST search was conducted for all sequences that met the threshold to remove possible redundancy. When a gene was represented by multiple sequences, the fold change with *q *value of only one sequence was selected to represent this gene.

### Real-time RT-PCR

Real-time RT-PCR analysis was carried out with the iQ SYBR Green Supermix kit (Biorad) using 200 nM of each amplification primer (see Additional file 2) and the 1^st^-strand cDNA (80 ng of the input total RNA equivalents) in a 25 μl reaction volume. The amplification was carried out on an iCycler iQ™ Real Time PCR Detection System (BioRad) with the following profile: 95°C – 60s; 40 cycles of 94°C-15s, 60°C -30s, and 72°C -30s. The melting curve analysis was performed for each primer pair. Expression levels of β-actin remained constant (within 0.5 Ct between samples) and were used as endogenous controls. Relative gene expression data were calculated using the 2^ΔΔ*C*T ^method [56].

## Authors' contributions

RWL conceived the study, carried out the microarray experiment, provided data analysis, and drafted the manuscript. CJL cultured the cells, carried out the western blotting and flow cytometry analysis, participated in data mining, and helped to draft the manuscript. Both authors read and approved the final manuscript.

## Supplementary Material

Additional file 1 Supplementary table 1. This data represents the 466 sequences identified to be significant at FDR = 0%, which represents 450 cattle genes. Click here for file

Additional file 2 Supplementary table 2. This data represents the forward/reverse primer sequences for the genes selected for real-time PCR confirmation. Click here for file

## Figures and Tables

**Figure 3:**
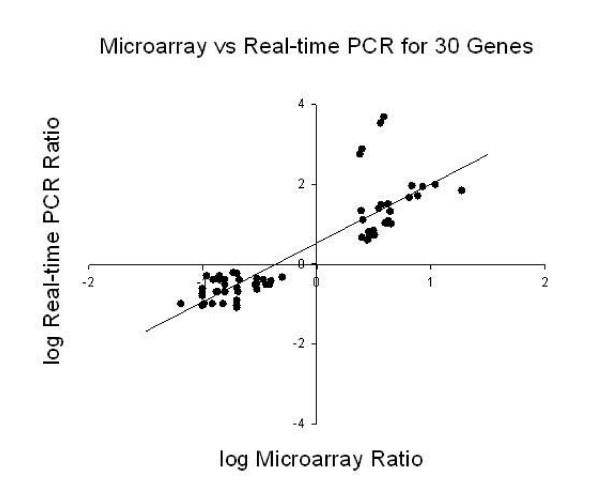
Correlation between levels of gene expression measured by microarray and real-time RT-PCR. The R = 0.867 (R^2 ^= 0.76) was calculated using log-transformed values of the fold change obtained for the 30 selected genes (n = 60).

**Table 1:** Cell cycle-related genes significantly regulated by butyrate.

Accession	Annotation	Fold	q-value*(%)
TC265170	Aurora kinase A (AURKA)	0.17	0
TC266385	Aurora kinase B (AURKB)	0.18	0
TC278061	Aurora-A kinase interacting protein (AURKAIP1)	0.19	0
TC281252	BUB1 budding uninhibited by benzimidazoles 1 homolog (BUB1)	0.10	0
TC281450	BUB1 budding uninhibited by benzimidazoles 1 homolog beta (BUB1B)	0.06	0
TC280420	CDC28 protein kinase regulatory subunit 2	0.02	0
TC292959	CDC2L1	0.04	0
TC293208	CDC45 cell division cycle 45-like	0.09	0
TC278776	Cell division cycle 2, G1 to S and G2 to M (CDC2)	0.12	0
TC285500	Cell division cycle 25A (CDC25A)	0.11	0
TC271610	Cell division cycle 25B isoform 3	0.14	0
TC294713	Cell division cycle 2-related	0.16	0
TC278478	Cell division cycle and apoptosis regulator 1	0.17	0
TC294633	Cell division cycle associated 2 (CDCA2)	0.08	0
TC292945	Cell division cycle associated 8	0.17	0
TC263741	Cell division cycle protein 20 homolog (p55CDC)	0.10	0
CK975861	Centromere protein F (350/400kD)	0.06	0
TC261165	Centromere protein-A (CENP-A)	0.09	0
TC267900	Centromeric protein E (CENP-E protein)	0.11	0
TC279457	CHK1 checkpoint homolog	0.16	0
TC293183	Chromatin assembly factor 1 subunit A (CAF-1)	0.20	0
TC268367	Chromobox protein homolog 5	0.10	0
TC297649	Chromodomain helicase DNA binding protein 3	0.14	0
TC265382	Chromosome condensation protein G	0.06	0
TC262952	Cyclin A2	0.17	0
TC265945	Cyclin B1 (CCNB1)	0.06	0
TC278429	Cyclin B2 (CCNB2)	0.22	0
TC262075	Cyclin G1	0.07	0
TC262855	Cyclin-dependent kinase 4	0.15	0
TC265450	Cyclin-dependent kinase inhibitor 2C	0.22	0
TC278545	Cyclin-dependent kinases regulatory subunit 1 (CKS-1)	0.09	0
TC284317	Defective in sister chromatid cohesion homolog 1	0.06	0
TC290880	Deoxythymidylate kinase (thymidylate kinase) (LOC506946)	0.15	0
TC273170	Discs large homolog 7 (DLG7)	0.22	0
TC265245	DNA directed RNA polymerase III polypeptide D	0.12	0
TC284361	DNA helicase (Blooms syndrome protein, RecQ protein-like 3)	0.11	0
TC267167	DNA mismatch repair protein Msh3 (mut S homolog 3)	0.24	0
TC278497	DNA polymerase epsilon p17 subunit gene	0.03	0
TC279954	DNA polymerase, alpha 2 (70kD subunit)(POLA2)	0.11	0
CK945922	DNA repair and recombination protein RAD54-likex	0.06	0
TC282404	DNA repair protein RAD51 homolog 1	0.11	0
TC290487	E2F transcription factor 4	0.11	0
TC282153	Establishment of cohesion 1 homolog 2	0.13	0
TC265789	Extra spindle poles like 1 (ESPL1)	0.08	0
TC291293	flap structure-specific endonuclease 1 (FEN1)	0.18	0
TC266990	G-2 and S-phase expressed 1	0.23	0
TC270253	GAJ protein (MND1)	0.07	0
TC292293	General transcription factor IIIC, polypeptide 2, beta 110kDa	0.24	0
BG688435	H/ACA ribonucleoprotein complex subunit 4 (Dyskerin, DKC1)	0.11	0
TC269917	Helicase, lymphoid-specific	0.13	0
TC267482	Histone deacetylase 8	0.15	0
TC276662	Histone H2A.Z	0.08	0
TC272233	Huntingtin interacting protein 2 (HIP2)	0.25	0
TC267608	Inner centromere protein	0.11	0
TC279078	Katanin p80 (KATNB1)	0.16	0
TC295304	Kinesin family member 11	0.23	0
TC294618	Kinesin family member 14	0.20	0
TC267353	Kinesin family member 20A	0.12	0
TC260588	Kinesin-like protein KIF22	0.09	0
TC291455	Kinesin-like protein KIF23 (Mitotic kinesin-like protein 1)	0.07	0
TC278220	Kinesin-like protein KIF2C (Mitotic centromere-associated kinesin)	0.19	0
TC266372	Kinetochore associated 2	0.15	0
TC284307	Lamin B1 (LMNB1)	0.05	0
TC277820	MAD2 mitotic arrest deficient-like 1	0.07	0
TC288554	Max interacting protein 1 (MXI1)	12.55	0
TC261106	MCM4 (CDC21 homolog) DNA replication licensing factor	0.14	0
TC264981	Minichromosome maintenance deficient 3 (MCM3)	0.10	0
TC277221	Minichromosome maintenance deficient 5 (MCM5, CDC46 homolog)	0.17	0
TC265572	Minichromosome maintenance deficient 6 (MCM6)	0.09	0
TC263581	Minichromosome maintenance protein 2	0.14	0
TC290639	Minichromosome maintenance protein 3 associated protein	0.16	0
TC293069	Mitotic spindle associated protein p126, MAP126	0.04	0
TC298939	M-phase phosphoprotein 1 (mitotic kinesin-like protein, MPHOSPH1)	0.13	0
TC260429	MutS homolog 6	0.21	0
TC266513	NEK2 (NIMA-related protein kinase 2)	0.13	0
TC278195	Nuclear ubiquitous casein and cyclin-dependent kinases substrate (P1)	0.19	0
TC279654	Nucleolar and spindle associated protein 1	0.08	0
TC274682	Nucleolin	0.10	0
TC293949	Origin recognition complex subunit 1	0.23	0
TC281941	PDZ-binding kinase (PBK)	0.15	0
TC278800	pescadillo homolog 1, containing BRCT domain (PES1)	0.17	0
TC289876	Proliferating cell nuclear antigen (PCNA)	0.09	0
TC263003	Proliferation-associated 2G4	0.16	0
TC276620	Proteasome (prosome, macropain) 28 subunit, alpha	0.21	0
TC260258	Proteasome subunit, beta type 8 (PSMB8)	0.14	0
TC292683	Protein C20orf172 (C20orf172)	0.06	0
TC300533	Protein regulator of cytokinesis 1	0.07	0
TC279370	Protein regulator of cytokinesis 1 (PRC1)	0.11	0
TC282034	RAD51 associated protein 1 (RAD51AP1)	0.21	0
TC280581	Replication factor C (activator 1) 3 (RFC3)	0.19	0
TC265859	Replication factor C (activator 1) 4 (RFC4)	0.03	0
TC263936	Ribonucleoside-diphosphate reductase M2 chain (RRM2)	0.17	0
TC275004	Scaffold attachment factor B2	0.20	0
TC266542	Sin3 associated polypeptide p30 (SAP30)	0.22	0
TC282970	SMC4 structural maintenance of chromosomes 4-like 1 (SMC4L1)	0.13	0
TC290192	Spindle pole body component 24 (kinetochore protein Spc24)	0.09	0
TC281963	Spindle pole body component 25 (kinetochore protein Spc25)	0.14	0
TC279153	Topoisomerase (DNA) II binding	0.05	0
TC278044	Transcription elongation regulator 1	0.09	0
TC281731	Transducin (beta)-like 1X-linked receptor 1 (TBL1XR1)	0.08	0
TC277942	Ubiquitin-conjugating enzyme E2 C (Ubiquitin-protein ligase C)	0.14	0
TC280380	Wee1-like protein kinase	0.16	0
TC279802	XPMC2 prevents mitotic catastrophe 2 homolog	0.21	0

* q-value is a percentage of a gene identified by chance as being significant.

**Table 2:** Apoptosis- and ECM-related genes significantly regulated by butyrate

Accession	Annotation	Fold	q-value (%)
CK975939	Tissue factor pathway inhibitor-2 (TFPI2)	0.07	0
TC266701	Fas apoptotic inhibitory molecule (FAIM)	0.07	0
CK965146	Collagen alpha 2(V) chain precursor (COL5A2)	0.12	0
TC281170	Tissue factor pathway inhibitor precursor (TFPI)	0.12	0
TC281607	Baculoviral IAP repeat-containing 5 (BIRC5)	0.16	0
TC261916	procollagen, type III, alpha 1 (COL3A1)	0.16	0
TC279824	spondin 1(SPON1)	0.19	0
TC289874	Insulin-like growth factor-binding protein 6 (IGFBP6)	0.2	0
TC275158	Insulin-like growth factor-binding protein 4 (IGFBP4)	0.21	0
TC265483	Programmed cell death 11(PDCD11)	0.27	0
TC276741	Insulin-like growth factor 2 (IGF2)	6.5	0
TC265993	Inhibin, beta A (INHBA)	9.48	0
TC277385	Adrenomedullin (ADM)	12.47	0
TC260960	Tissue inhibitor of metalloproteinase 2 (TIMP2)	14.05	0

**Table 3:** Comparison of gene expression levels between microarray and real-time RT-PCR

Accession	Annotation	Microarray*	Real-time PCR**
TC281450	BUB1 budding uninhibited by benzimidazoles 1 homolog beta (BUB1B)	0.1	0.1
TC266701	Fas apoptotic inhibitory molecule (FAIM)	0.1	0.3
TC277820	MAD2 mitotic arrest deficient-like 1 variant (MAD2L1)	0.1	0.1
TC261165	Centromere protein A (CENPA)	0.1	0.2
TC289876	Proliferating cell nuclear antigen (PCNA)	0.1	0.1
TC290192	Spindle pole body component 24 (SPBC24)	0.1	0.2
TC269605	Hepatocyte growth factor (scatter factor, HGF)	0.1	0.4
TC267353	Kinesin family member 20A (KIF20A)	0.1	0.1
TC260258	Proteasome subunit, beta type 8 (PSMB8)	0.1	0.2
TC262855	Cyclin-dependent kinase 4 (CDK4)	0.1	0.6
TC290639	Minichromosome maintenance protein 3 associated protein (MCM3AP)	0.2	0.5
TC289874	Insulin-like growth factor-binding protein 6 (IGFBP6)	0.2	0.3
TC262471	Heat shock 90kD protein 1, alpha (HSP90AA1)	0.2	0.5
TC295998	Activator of S phase kinase	0.3	0.3
TC277755	CSE1 chromosome segregation 1-like (CSE1L)	0.3	0.4
TC261112	Profilin 1 (PFN1)	0.4	0.4
TC294142	β-catenin (CTNNB1)	1.4	5.8
TC260308	GM2 ganglioside activator protein (GM2A)	2.3	6.6
TC267034	similar to LIM domain containing 2 (LIMD2)	2.3	4.2
TC292975	unknown	2.5	17.1
TC276824	Dual specificity protein phosphatase 1 (MKP-1)	2.8	6.6
TC260543	Cathepsin D (CTSD)	3.0	5.1
TC277986	Cathepsin L precursor (CTSL)	3.2	2.7
TC276574	Protease, serine, 11 [IGF binding] (PRSS11)	3.8	11.2
TC298909	Serine/threonine kinase 32C (STK32C)	4.3	26.2
TC265800	Activating transcription factor 3 (ATF3)	4.5	10.2
TC277745	CITED2	6.3	47.6
TC276741	Insulin-like growth factor 2 (IGF2)	6.5	100.0
TC280815	Matrix metalloproteinase 13 (MMP13)	8.3	87.8
TC265993	Inhibin, beta A (INHBA)	9.5	82.8

* fold change (microarray) = mean background-adjusted, normalized signal intensity from the butyrate treated group divided by mean background-adjusted, normalized signal intensity from the control group.

** fold change (real-time PCR): expression in the control group was set at 1.0. The values below 1.0 indicated down-regulation by butyrate.
